# The Clinical Evaluation of Alcohol Intoxication Is Inaccurate in Trauma Patients

**DOI:** 10.7759/cureus.2190

**Published:** 2018-02-14

**Authors:** Ashwini Kumar, Travis Holloway, Stephen M Cohn, Gregory Goodwiler, John R Admire

**Affiliations:** 1 Surgery, University of Texas Health Science Center at San Antonio; 2 Surgery, Staten Island University Hospital

**Keywords:** alcohol, alcohol metabolism, breathalyzer, elimination of ethanol, intoxication, trauma

## Abstract

Background: Discharging patients from emergency centers based on the clinical features of intoxication alone may be dangerous, as these may poorly correlate with ethanol measurements.

Objective: We determined the feasibility of utilizing a hand-held breath alcohol analyzer to aid in the disposition of intoxicated trauma patients by comparing serial breathalyzer (Intoximeter, Alco-Sensor FST, St. Louis, Missouri, USA] data with clinical assessments in determining the readiness of trauma patients for discharge.

Methods: A total of 20 legally intoxicated (LI) patients (blood alcohol concentration (BAC) >80 mg/dL) brought to our trauma center were prospectively investigated. Serial breath samples were obtained using a breathalyzer as a surrogate measure of repeated BAC. A clinical exam (nystagmus, one-leg balance, heel-toe walk) was performed prior to each breath sampling.

Results: The enrollees were 85% male, age 30±10 (range 19-51), with a body mass index (BMI) of 29±7. The average initial body alcohol level (BAL) was 245±61 (range 162-370) mg/dL. Based on breath samples, the alcohol elimination rates varied from 21.5 mg/dL/hr to 45.7 mg/dL/hr (mean 28.5 mg/dL/hr). There were no significant differences in alcohol elimination rates by gender, age, or BMI. The clinical exam also varied widely among patients; only seven of 16 (44%) LI patients demonstrated horizontal nystagmus (suggesting sobriety when actually LI) and the majority of the LI patients (66%) were able to complete the balance tasks (suggesting sobriety).

Conclusion: Intoxicated trauma patients have an unreliable clinical sobriety exam and a wide range of alcohol elimination rates. The portable alcohol breath analyzer represents a potential option to easily and inexpensively establish legal sobriety in this population.

## Introduction

A high percentage of adults presenting to level I trauma centers are under the influence of alcohol (as high as 50% at one center (unpublished data). The optimal method to determine sobriety and readiness for discharge in patients is uncertain. Some institutions utilize a nomogram based upon a presumed clearance rate to estimate when the patient’s serum alcohol level will decrease to the legal limit. Unfortunately, the alcohol metabolism rate varies significantly between individuals, ranging from 13 to 25 mg/dL/h in nonalcoholics and 30 to 50 mg/dL/h in alcoholics [1-9]. Other centers rely on a clinical assessment to determine sobriety. After the patient is judged sober, a tertiary exam is done and the patient is discharged home. The clinical features of intoxication may poorly correlate with the ethanol level [9] and, therefore, discharging patients from the ER based on clinical features can be dangerous. Thus, the optimum time for discharging intoxicated patients from the ER has been a subject of debate. Keeping these patients in the ER for prolonged periods comes at the expense of the hospital provider’s time, hospital resources, and bed occupancy. On the other hand, the premature discharge of inebriated patients can result in liability for the institution when patients cause harm to themselves or others [10]. All this confusion regarding attaining sobriety in the intoxicated trauma patient creates an undue burden in our already overcrowded Emergency Centers.

We sought to compare serial breathalyzer (Intoximeter, Alco-Sensor FST, St. Louis, Missouri USA) data (sobriety defined as a blood alcohol concentration (BAC) < 80 mg/dL) with a clinical assessment in determining the readiness of trauma patients for discharge. Our aim was to determine the feasibility of utilizing a hand-held breath alcohol analyzer to aid in the disposition of intoxicated trauma patients.

## Materials and methods

Our study protocol was developed at University Hospital, a level I trauma center in San Antonio, Texas, and was approved by the institutional review board (IRB) at the University of Texas Health Science Center. A total of 20 legally intoxicated trauma patients were prospectively enrolled. The patients were approached if they had sustained minimal injury based upon the initial clinical exam and imaging and were unlikely to require admission to the hospital. These patients remained in the emergency department for the entirety of their study involvement. This study did not represent a change in practice; therefore, some of the patients were ultimately admitted for injuries discovered on the tertiary exam or while waiting to become legally sober. An initial determination of legal intoxication was made with the measurement of the blood alcohol concentration (BAC) upon arrival. This is the current standard of care in the trauma population at this emergency department when there is suspicion of intoxication. A BAC greater than 80 mg/dL (0.08 g/dL) was considered legally intoxicated (LI).

Breathalyzer testing

After the baseline BAC was ascertained, serial breath analyzer testing was performed using an Intoximeter Alco-Sensor FST (cost = 40 cents/sample). The first breath sample was taken at half the estimated time it takes the individual to eliminate alcohol to the point where the blood alcohol level is below the legal limit (This estimation was based on previously published average clearance rates (20.43 mg/dL/hr)) [8]. Subsequent to the initial breath analysis, serial breath tests were performed at two-hour intervals until a breathalyzer sample was obtained that was below 80 mg/dL.

Clinical testing

Prior to each breath sample obtained, a three-part clinical exam was administered to assess clinical sobriety. Horizontal nystagmus was tested by placing a finger approximately one foot in front of the patient and moving the finger slowly but steadily horizontally across the patient’s field of vision. The examiner instructed the patient to follow the movement with their eyes and recorded a positive horizontal nystagmus test if one of the following was met: lack of smooth tracking of the finger, gross nystagmus at the maximal lateral position of the patient’s gaze, or the manifestation of nystagmus prior to 45 degrees. Second, a heel-to-toe walking test was performed where patients were instructed to walk the length of their exam room, approximately 15 feet on a line. A positive heel-to-toe walk test was recorded if the patient was unable to walk the complete distance of the line, the patient missed the line during their progression, or there were extreme shifts in balance. Finally, a one-leg balance test was performed where patients were asked to stand on one leg for 10 seconds and repeated using the other leg for an additional 10 seconds. A positive test was recorded if the patient was unable to maintain balance on one leg for the entire preset time or if they had to steady themselves to prevent falling.

A determination of LI was made based on breath analysis results. Once a subject was below the legal limit of alcohol on breath analysis, the clinical testing of intoxication and breath analysis was discontinued and the patient was deemed to have completed the study. All patients were approached again following the conclusion of the study, to ensure there were no questions or changes in consent in the study.

Data analysis

All patients that required trauma activation were screened for enrollment during the time when research personnel were present. The potential subjects were approached after the conclusion of the primary survey and any initial imaging. The clinical team gave their initial assessment to the research team as to the injuries sustained, initial body alcohol level (BAL), and the likelihood of admission for the injuries sustained. A clinical data sheet was created for each subject. The completion of the clinical examination was recorded as “positive,” “negative,” or “unable to complete.” A breath analysis sample was obtained at the same time as the clinical exam and recorded in mg/dL.

**Figure 1:**
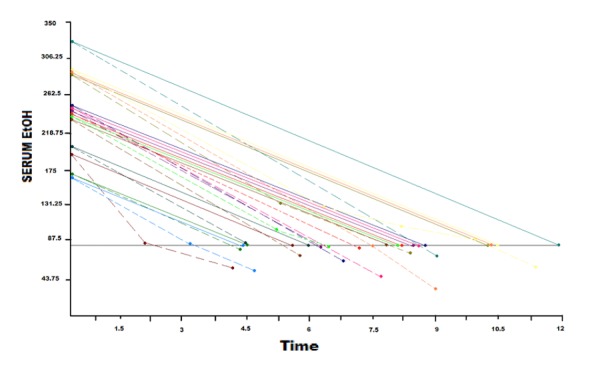
Dashed lines represent breath sample measurements, solid lines represent a standard elimination rate of 20 mg/dl/hr Different colors represent the individuals.

Using the breath analysis values, a linear slope or best-fit line was determined for each individual (dashed lines) and it was compared to the conservative elimination rate of 20 mg/dL/hr (solid lines). The slope of this line represented the average clearance rate (Figure 1). In addition, the likely time from initial BAL until ethanol clearance was calculated using the previously published 20 mg/dL/hr [8]. This time was compared to the time it took for a breath value to reach a level below 80 mg/dL. In addition, subjects were divided by age, gender, and BMI to determine any association with clearance rates. 

The Clinical exam results were placed in three separate 2x2 squares based on the breathalyzer results at the time of that exam. These were analyzed for association.

## Results

Eighty-five percent of the enrollees were male. The average age of the subjects was 30 (range 19-51 years). The average BMI was 28.9, with a standard deviation of 7.1. The average initial BAL was 245 ± 61 (range 162-370) mg/dL. Based on breath samples, the alcohol elimination rates varied from 21.5 mg/dL/hr to 45.7 mg/dL/hr (average 28.5 mg/dL/hr). When examining the patients in groups based on gender, age, and BMI, there were no statistically significant differences in the elimination rates of alcohol.

The clinical exam varied widely among patients. There was a lack of correlation between breath ethanol (EtOH), nystagmus, balance, and heel-toe tests (Table 1). Of the 16 patients who were able to participate in the horizontal nystagmus test, only seven (44%) demonstrated a positive horizontal nystagmus. This suggests clinical sobriety; however, these patients continued to have elevated breath alcohol levels (> 80 mg/dL). A total of 66% of the patients who were able to complete the balance tests passed them, despite having breath alcohol levels > 80 mg/dL. Also, of the five patients able to participate, four intoxicated patients (80%) were able to successfully perform the heel-toe test. This suggests clinical sobriety when these patients continued to have elevated breath alcohol levels and, therefore, none of the clinical exams are sensitive or specific for determining breath EtOH levels and degree of intoxication (Table 1).

**Table 1:** The three clinical exams conducted were for the presence of nystagmus, ability to balance on one leg, and ability to walk heel to toe. The ability to conduct each exam did improve when patients reached legal sobriety (EtOH EtOH: breath ethanol

Clinical exam	EtOH level > 80 mg/dl	EtOH level < 80 mg/dl
Nystagmus (+)	7	9
Nystagmus (-)	6	9
Unable to complete exam	7	2
Total	20	20

Clinical exam	EtOH level > 80 mg/dl	EtOH level < 80 mg/dl
One leg balance (+)	4	9
One leg balance (-)	3	6
Unable to complete exam	13	5
Total	20	20

Clinical exam	EtOH level > 80 mg/dl	EtOH level < 80 mg/dl
Heel-toe walk (+)	4	8
Heel-toe walk (-)	1	2
Unable to complete exam	15	10
Total	20	20

## Discussion

Alcohol ingestion directly affects risk-taking behavior in humans [11]. It has been proven that there is a time-course effect of alcohol dose on attention span, impulsivity, discrimination, and response time [12]. The optimum time for discharging intoxicated patients from the ER remains a subject of considerable debate. In our study, we found that alcohol elimination rates varied widely (21.5 mg/dL/hr to 45.7 mg/dL/hr) among our trauma patients and that clinical examination was completely unreliable in predicting blood alcohol concentration.

Enzymatic oxidation in the liver is the principal path of elimination of alcohol; a small quantity of unmetabolized alcohol is excreted in the urine, breath, and perspiration [13]; which permits alcohol concentration to be measured in breath and urine. Quantitative data on alcohol elimination has been obtained, mostly from the repeated measurement of blood or breath alcohol concentrations, but a range of clearance rates have been reported [9,12].

Currently, the criteria for discharging intoxicated patients vary between institutions. While some rely on clinical evaluation for this purpose, others wait for the alcohol level to fall under legal limits. Clinical suspicion begins with the measurement of BAC during the initial exam and if injuries are minor, alcohol concentration, along with clinical examinations, are consecutively performed until they reach safe levels prior to discharge. Unfortunately, the results of clinical examination appear poorly correlated with the actual intoxication of the patient [9] and without adjuvant objective data, can overestimate the level of sobriety in this population. Discharging patients from the ER based on clinical judgment alone can also lead to errors in management, placing patients at further risk of injury.

We are often faced with difficulties relating to patient overflow in our emergency centers and trouble with the disposition of patients after minor trauma. This raises the question of how to properly manage patients who are legally intoxicated and awaiting sobriety in our ED and trauma departments. With the wide range of clearance rates and no universal consensus, some patients are receiving multiple blood draws, which increases the cost of care and the unnecessary utilization of resources. The time factor is also considerable during the clearance in these patients; while awaiting definitive management, some patients are inappropriately admitted in order to free up space in the ED.

To mitigate risk, cost, and time management, we have found that a breathalyzer is both clinical and cost-effective. Despite the relatively high initial cost (USD 555), it can be used repeatedly and reduce the cost of care without affecting quality. It can be utilized in the ED with very little training required, and the results are immediate. Breathalyzers are commonly used in the field by law enforcement but can be utilized in the hospital setting as an ancillary diagnostic tool along with clinical evaluation.

Limitations

Although the breathalyzer is easy to operate, appropriate training needs to be provided to emergency room staff. The breathalyzer should be calibrated every six months to ensure appropriate functioning; otherwise, the machine may give faulty readings. Also, alcohol absorption may vary from a few minutes to over two hours after consumption, depending on the type and amount of food ingested. These factors should be taken into account while performing the breathalyzer test.

## Conclusions

Given the wide range of alcohol elimination rates and the inaccuracy of clinical exams, a portable alcohol breath analyzer has the potential to establish legal sobriety in the trauma population. This instrument is already being used by law enforcement, is readily available, easy to use, and inexpensive.

## Appendices

1. Why is this topic important?

The results of clinical examinations are poorly correlated with the actual intoxication of the patient, and some patients receive multiple blood draws, which only amounts to an increased cost of care and the unnecessary utilization of resources. Discharging patients from the ER based on clinical judgment alone can also lead to errors in management, placing patients at further risk of injury.

2. What does this study attempt to show?

The breathalyzer is both clinical and cost-effective. It can be utilized in the ED with very little training. Breathalyzers are commonly used in the field by law enforcement but can be utilized in the hospital setting as an ancillary diagnostic tool along with clinical evaluation.

3. What are the key findings?

There were no statistically significant differences in the elimination rates of alcohol when examining patients in groups based on gender, age, and BMI. None of the subjects metabolized EtOH slower than 20 mg/dL/hr. Also, the clinical exam varied widely among patients. In addition, a breathalyzer is both clinical and cost-effective.

4. How is patient care impacted?

The use of breathalyzers can help determine the appropriateness of discharge timing for inebriated patients and can reduce the cost of care, without affecting quality.
